# A Weakly-Supervised Named Entity Recognition Machine Learning Approach for Emergency Medical Services Clinical Audit

**DOI:** 10.3390/ijerph18157776

**Published:** 2021-07-22

**Authors:** Han Wang, Wesley Lok Kin Yeung, Qin Xiang Ng, Angeline Tung, Joey Ai Meng Tay, Davin Ryanputra, Marcus Eng Hock Ong, Mengling Feng, Shalini Arulanandam

**Affiliations:** 1 Saw Swee Hock School of Public Health, National University of Singapore, Singapore 117549, Singapore; ephwha@nus.edu.sg; 2 Singapore Civil Defence Force, Singapore 408827, Singapore; wesley_yeung@nuhs.edu.sg (W.L.K.Y.); ng_qin_xiang@scdf.gov.sg (Q.X.N.); Angeline_tung@htx.gov.sg (A.T.); tay_ai_meng@scdf.gov.sg (J.A.M.T.); Davin_RYANPUTRA@nuhs.edu.sg (D.R.); Shalini_ARULANANDAM@scdf.gov.sg (S.A.); 3 National University Hospital, National University Health System, Singapore 119074, Singapore; 4 Home Team Science & Technology Agency, Singapore 329560, Singapore; 5 Health Services Research Centre, Singapore Health Services, Singapore 169856, Singapore; marcus.ong.e.h@singhealth.com.sg; 6 Health Services and Systems Research, Duke-NUS Medical School, National University of Singapore, Singapore 169857, Singapore; 7 Department of Emergency Medicine, Singapore General Hospital, Singapore 169608, Singapore; 8 Institute of Data Science, National University of Singapore, Singapore 117602, Singapore

**Keywords:** emergency medical services, clinical audit, natural language processing, named entity recognition, weakly-supervised learning

## Abstract

Clinical performance audits are routinely performed in Emergency Medical Services (EMS) to ensure adherence to treatment protocols, to identify individual areas of weakness for remediation, and to discover systemic deficiencies to guide the development of the training syllabus. At present, these audits are performed by manual chart review, which is time-consuming and laborious. In this paper, we report a weakly-supervised machine learning approach to train a named entity recognition model that can be used for automatic EMS clinical audits. The dataset used in this study contained 58,898 unlabeled ambulance incidents encountered by the Singapore Civil Defence Force from 1st April 2019 to 30th June 2019. With only 5% labeled data, we successfully trained three different models to perform the NER task, achieving F1 scores of around 0.981 under entity type matching evaluation and around 0.976 under strict evaluation. The BiLSTM-CRF model was 1~2 orders of magnitude lighter and faster than our BERT-based models. Our proposed proof-of-concept approach may improve the efficiency of clinical audits and can also help with EMS database research. Further external validation of this approach is needed.

## 1. Introduction

Clinical performance audits are thought to be an important part of quality review and continuous quality improvement in healthcare systems and services [1,2]. In emergency medical services (EMS), one of the clinical audits that is conducted involves examining whether paramedics have performed the assessment and treatment steps following the standard operating procedures [3,4,5]. This is usually performed by auditing the free text reports written by the paramedics for the attended cases.

EMS clinical audits need to be routinely performed to ensure adherence to treatment protocols, to identify lapses, and to discover systemic deficiencies to guide paramedic training. However, identification of these items for audit from the free text case reports requires a significant amount of time, resources, and effort [6].

The Singapore Civil Defence Force (SCDF) is the national emergency medical services (EMS) provider in Singapore, handling more than 190,000 medical calls to the national “995” emergency hotline annually [7]. Paramedics in the SCDF are trained to respond to medical emergencies by providing rapid on-scene triage, treatment, and conveyance of casualties to the hospitals for further management. At present, all EMS-attended cases are recorded by the paramedics on hardcopy ambulance case records, then transcribed into electronic forms (with the assistance of a digital pen) within 48 h and uploaded onto an internal server for audit and data analysis. Clinical audit of our EMS involves a manual, laborious audit of randomly selected cases and subsequent follow-up actions by a considerably small team of dedicated auditors. Due to the high call volume and inherent complexity of the paramedic protocols, only a limited percentage (around 10%) of total cases were audited every year [8].

Named entity recognition (NER) is a natural language processing (NLP) technique that recognizes and labels certain words mentioning specific entities in the sentences. An example of NER is to recognize “Apple” as a brand name in the sentence “I am a big fan of Apple products” but not “Apple is my favorite fruit”. It has been successfully used for information extraction in medical texts [9,10], but its specific application in the context of paramedic text reports is unexplored. The language used in the paramedic text reports are different from that in traditional clinical documents, so existing non-trainable NER models cannot be directly applied in this case. Challenges that are common for clinical NLP still apply, which include widespread and inconsistent use of acronyms, misspellings, flexible formatting, atypical grammar and use of jargon [11]. Lastly, it is impractical to label a large amount of corpus to generate a dataset for fully supervised training.

In this proof-of-concept study, we aimed to develop an NER model on paramedic text reports for clinical audit. We adopted a weakly-supervised approach by creating and fine-tuning a synonym list of keywords and phrases for the entities and using them as the pseudo labels. The main contribution of the paper is to (1) propose the use of natural language processing to conduct EMS clinical audits instead of human chart reviewing, (2) use a weakly-supervised method to label a mass amount of unlabeled data for downstream training and (3) ascertain the effectiveness of the method in EMS clinical audit data. Since the languages used in different EMS systems are dramatically different, we hoped that this study could serve as a good example for other EMS systems to develop their own clinical NER audit model.

The remainder of this paper is structured as follows. In the Methods section, we introduce the steps of (1) data preparation and preprocessing, (2) weakly-supervised labelling, (3) model training and (4) model evaluation. A web demo of the model is also developed. In the Results section, we describe the dataset and report the performance, size and inference speed of the models. In the Discussion section, we discuss our approach, with its advantages and limitations, and analyze the errors.

## 2. Methods

### 2.1. Data Preparation and Preprocessing

The data used in this proof-of-concept study contained 58,898 ambulance incidents recorded by the Singapore Civil Defence Force between 1st April 2019 and 30th June 2019. We included all incidents from one of the three clinical scenarios that are commonly encountered in EMS practice as motivating examples: (1) acute coronary syndrome, (2) stroke, and (3) the bleeding patient. After excluding 14,679 incidents that did not result in a patient encounter and 8 cases with missing text reports, the remaining 44,211 incidents were used as the final dataset. Ethics approval for this study was granted by the National Healthcare Group (NHG DSRB 2020/00893) with a waived informed consent.

Text reports were converted into lower case as many text reports were entered in all upper case due to the nature of the data entry system. All symbols were removed except for the “%” symbol, while all numbers were retained. Sentences were split into individual tokens using white space tokenization. All preprocessing steps were performed automatically by a simple Python script with native libraries and regular expressions.

### 2.2. Weakly-Supervised Labelling

As the entire dataset was unlabeled, we used a weakly-supervised learning approach to model training. For the NER labels, we chose 17 different clinical entities spanning 3 different categories (clinical procedure, clinical finding, and medication) based on their clinical relevance in EMS practice. The notation used in this study is the IOB2 notation, which assigns a “B-” token for the first word of each entity, including terms which comprise of only 1 word; an “I-” token for each subsequent word within the entity; and an “O” token for all other tokens not belonging to any entity [12]. We did not annotate negation because we consider the paramedics to be compliant if they documented the entity. Documentation indicated that they did not forget about the standard operating procedures and may have chosen to overwrite according to the actual scenario.

In the first step, we used a rule-based technique to create dummy labels for the entire dataset. We first created a list of synonyms for each entity based on EMS practice experience. These synonyms might be single token or multiple tokens, and each entity can have multiple synonyms. Subsequently, we used the synonyms to match and pseudo-label the entities in the sentences. Fuzzy string matching was used to increase recall of the bootstrapping process by including terms with minor spelling mistakes, defined as having a maximum of 1 missing character compared to the correct spelling. However, as this might result in high rates of false positives if used on short phrases, it was only performed on strings that were 5 characters or longer. The fuzzy string-matching algorithm used was provided by the “fuzzysearch” package [13]. Single or multi-token entities that spanned less than 5 characters were matched using exact string matching. If explicitly specified, exact string matching was used.

After pseudo-labelling of the training, development and test sets, the labels in the development and test sets were verified by a clinician and any mistakes made by the bootstrap process were corrected. We performed error analysis only on the development set to fine-tune the synonym list and specify which terms require exact string matching to improve the labelling process. The dataset was split into 95% training (*n* = 42,000), 2.5% development (*n* = 1105) and 2.5% test (*n* = 1106) sets. Only 5% (*n* = 2211) of the data were human labelled.

### 2.3. Model Training

To perform the NER task, we experimented with a deep learning-based Bidirectional Long Short-Term Memory + Conditional Random Fields (BiLSTM-CRF) model as well as two Bidirectional Encoder Representations from Transformer (BERT) models with different pretrained weights. Both model architectures could automatically learn the useful information from the training data without manual feature engineering.

#### 2.3.1. Bidirectional Long Short-Term Memory + Conditional Random Fields

**Figure 1:**
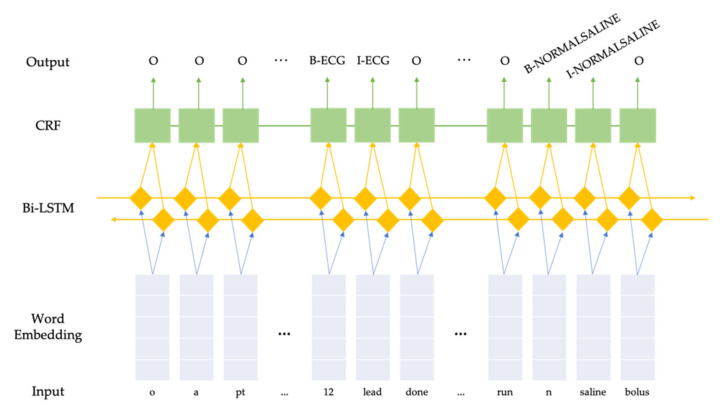
Illustration of our BiLSTM-CRF model.

Conditional Random Fields (CRF) are a class of probabilistic models designed to segment and label sequence data [14], and have been used with success on named entity recognition tasks due to their ability to use customized observation features from both past and future elements in sequences [15]. Bidirectional Long Short-Term Memory [16] combined with an output CRF layer [17] is a recurrent neural network (RNN) model that has achieved state-of-the-art performance over many named entity recognition tasks. Instead of manually crafting features for the traditional CRF model, a BiLSTM model automatically learns the useful features and feeds them into the CRF model. We built a BiLSTM-CRF model using PyTorch library in Python [18]. No pre-trained word embedding was used; instead, a word embedding layer was initialized with 0 s and trained together with the entire model. The batch size was set as 512. We used Adam optimizer [19] with a default learning rate of 0.001. Early stopping was implemented and would trigger if the validation loss did not decrease in 5 consecutive epochs to prevent overfitting. The maximum training epochs was set as 300. We experimented with different dimensions of the word embedding layer as well as the hidden layer, and used 100 and 64, respectively, in the final model, which yielded the best performance on the validation set. An illustration of the model can be seen in Figure 1.

#### 2.3.2. BERT-Based Token Classifier

**Figure 2:**
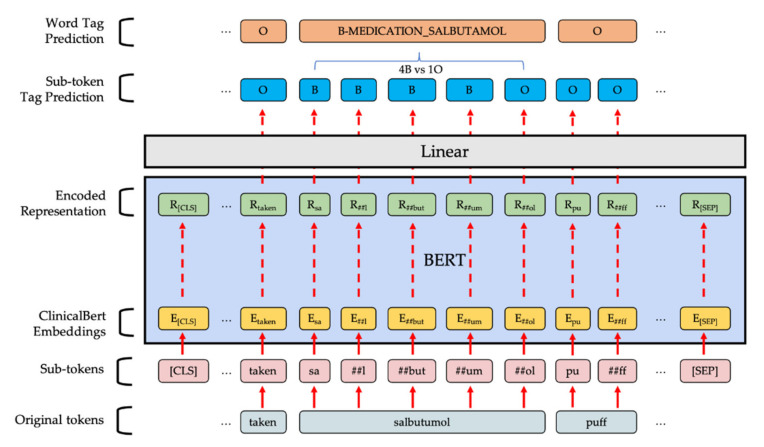
Illustration of our BERT-based token classification model. Sub-tokens with ## as prefix are split from original tokens and will have different embeddings compared to the ones with same spelling but without ##.

BERT-based models are bidirectional transformer models with contextualized word embedding pre-trained on large corpora and have revolutionized deep learning in NLP tasks ever since their introduction [20]. Token classification can be achieved by adding a linear classification layer after the output from the BERT-based model. To build the model, we used the PyTorch implementation from the Transformers Python library by HuggingFace [21]. The first pretrained model we used was the *BERT-based-uncased* model, which was trained on two large corpora: BooksCorpus [22] and English Wikipedia. Since our corpus is related to the clinical domain, the second pretrained model we used was Clinical-BERT [23], which was trained on clinical notes from the Medical Information Mart for Intensive Care III database [24]. Prior to the training, all sentences were tokenized by the pre-trained tokenizer and zero-padded to a constant 300 token sequence length. In the training phase, the pre-trained model was fine-tuned on our training data for 30 epochs with early stopping after 5 epochs if there was no improvement in token level accuracy on the development set. We used the AdamW optimizer [25] under the learning rate of 3 × 10^−5^, Adams epsilon of 1 × 10^−8^ and weight decay rate of 0.01 over all parameters except for the bias terms as well as the gamma and beta terms in the layer-normalization layers. A learning rate scheduler was used to linearly reduce the learning rate throughout the epochs. When the model combined the sub-token tag predictions, we let the model pick the most frequent class except O to be the final prediction of the word. An illustration of the model can be seen in Figure 2.

### 2.4. Model Evaluation

#### 2.4.1. Token Class Level

We evaluated the performance of our NER models using the weighted precision, recall and F1-score on all tokens except the uninformative “O” token. Specifically, the weighted metric calculates the metric for each token class and finds their average weighted by the number of true tokens in that class.

#### 2.4.2. Entity Level

We reported the MUC-5 evaluation metrics under both strict evaluation mode and entity type matching mode defined in the 2013 International Workshop on Semantic Evaluation (SemEval’13) to compare their performance at the entity level [26,27]. An entity prediction is defined by both the entity type predicted and the word span (starting word and ending word). MUC-5 categorizes each prediction into 1 of the 5 following types:

- Correct (COR): the system’s output is the same as the gold-standard annotation.
- Incorrect (INC): the system’s output has nothing in common with the gold-standard annotation.
- Partial (PAR): the system’s output shares some overlapping text with the gold-standard annotation.
- Missing (MIS): a gold-standard annotation is not captured by the system.
- Spurius (SPU): the system labels an entity which does not exist in the gold-standard annotation.

Based on these types, the below measures can be calculated:

- Possible (POS): the number of annotations in the gold-standard which contribute to the final score.

POS = COR + INC + PAR + MIS = True Positive (TP) + False Negative (FN)

- Actual (ACT): the total number of annotations produced by the system.

ACT = COR + INC + PAR + SPU = True Positive (TP) + False Positive (FP)

- Precision: the percentage of entities found by the system that are correct.

P = COR/ACT = TP / (TP + FP)

- Recall: the percentage of entities present in the data that are found by the system.

R = COR/POS = TP / (TP + FN)

- F1-score: the harmonic mean of precision and recall.

F1 = 2 × P × R / (P + R)

Under both strict evaluation mode and entity type matching mode, there will be no PAR. The only difference between the two modes is that if a predicted entity has the correct type but the word span only overlaps with the gold-standard annotation, it will be INC under strict evaluation, but COR under entity type matching. It is worth noting that POS depends on the model-specific prediction and can be larger than the total number of entities in the data, because 1 gold-standard entity can be compared to more than 1 prediction overlapped with it and, thus, be counted more than once.

### 2.5. Web Demo

We built a publicly accessible website (https://emsnlp.herokuapp.com/, accessed on 1 July 2021), with the Flask web application framework [28], Jinja2 template engine [29], and Heroku cloud application platform [30]. Visualization of the predicted entities was performed using displaCy [31].

## 3. Results

The whole dataset consists of 44,211 paramedic reports, with 3,069,578 words and 39,067 unique words. The training, development and test sets contained 41,984, 1105 and 1106 reports, respectively. Table 1 shows some examples of the original reports, the reports after preprocessing and their ground truth NER labels. Statistics about the clinical entities, their relative frequencies, total tokens of the entities and average number of tokens per entity are presented in Table 2.

Based on the prevalence of the entities, Electrocardiogram (ECG), Bleeding and Stroke Assessment are the three most observed entities. Looking at the tokens, *Normal Saline* has a significantly longer average number of tokens per entity, because it is often represented by phrases such as “i v n s” or “iv ns 0 9%” after the punctuation is removed.

Table 3 shows the performance of our NER models over the entities on the test set. On the test set, our models show indistinguishably excellent performance of F1 scores: around 0.981 under entity type matching evaluation and 0.976 under strict evaluation.

Despite the indistinguishable performance, the model complexity and inference speed differ by 1~2 orders of magnitude between the BiLSTM-CRF model and the BERT-based models, as demonstrated in Table 4. Hence, we decided to choose BiLSTM-CRF as our final model. We reported the performance of the BiLSTM-CRF model over the token classes on the development set and test set in the supplementary file.

## 4. Discussion

In this proof-of-concept study, we developed an NER model on paramedic text reports for the purpose of clinical audit. Although not quantified in this study, it is apparent that this system would enable us to vastly increase not only the number of cases audited, but also the complexity of the audit, as dozens of individual actions could be evaluated. Moreover, this could be achieved in a much shorter period of time than if a team of human auditors were to perform manual chart reviews.

Another possible use case for this model would be to identify cases for database research. With the digitalization of ambulance data, there is increasing opportunity for large-scale data analysis and research. The NER model was able to accurately identify a limited set of commonly used clinical entities. This information can be used to retrieve cases containing a certain clinical entity. This would reduce the need for a manual chart review, which is impractical as the number of cases vastly increases the potential sample size for any clinical study. As EMS is an important part of the chain of survival for patients requiring emergency care, there is a need for robust identification of case types for downstream research tasks.

The final BiLSTM-CRF model achieved good performance with an F1 score of 0.981 under entity type matching evaluation and 0.976 under strict evaluation. Although the overall performance was satisfactory, we observed two major sources of errors. The first source is a partial capture of the full span of multi-word entities including ECG (e.g., “ecg 4 leads”), GTN (e.g., “s l gtn”) and Normal Saline (e.g., “i v n s 0 9%”). Since our training set is labelled only via a weakly-supervised approach, while the validation set and test set are labelled by humans, slight discrepancies are expected. Nevertheless, we believe these mistakes are of lesser significance and would not affect the audit result since the entities are still labelled. The second source is due to the misspelled words. A misspelled word can either be non-existent (“salbutumol” vs. “salbutamol”) or have a different meaning (“facial drop” vs. “facial droop”). Our BiLSTM-CRF model will mark the first type of misspelled words as “unknown” where a special word vector will be assigned. As for the second type of misspelled words, they are seen both in their normal context and the misspelled context. As a result, these misspelled words are more difficult to learn for the model and contribute to the errors. With that being said, we also observed that some of these misspelled words were correctly predicted, likely thanks to the CRF module.

We expected the BERT models to mitigate the issue of misspelling by predicting the entities from the subwords produced by WordPiece tokenization [32]. Moreover, we expected Clinical-BERT would perform better with the pretraining on a clinical corpus. Upon examination of the results, we found that pretrained tokenizers from BERT_base_ and Clinical-BERT did produce different subwords. Based on these subwords, the BERT models predict more entities than the BiLSTM-CRF model. However, both true positive and false positive results increased, and we observed higher recall, lower precision and F1 scores similar to the BiLSTM-CRF model. Reasons why the BERT models may have not worked better than BiLSTM-CRF in our task include the following: (1) the WordPiece tokenization is not designed to correct spelling errors, but rather to segment the meaningful units from the words; (2) in our paramedic report, the words are highly abbreviated, making the tokenizer less helpful; and (3) clinical notes, which Clinical-BERT was pre-trained on, are indeed different from our paramedic report to a certain extent.

To our knowledge, this is the first study investigating the use of NLP on a large number of paramedic-written free text reports, and the results are promising. We believe that this work can inspire more NLP applications for novel clinical text. Nonetheless, we also recognize some limitations of this work. Firstly, the study was conducted within a single EMS system and further studies are needed to evaluate its external validity. Further planned intervention is also necessary to evaluate the usefulness of this system. Secondly, we did not manage to correct the spelling errors in the text reports, which is an area for future work. Thirdly, we did not experiment with lighter BERT-based models, such as DistilBERT, which are smaller and faster than normal BERT models [33]. Lastly, entity classes such as “Burns Cooling” and “Valsalva Maneuver” were absent in the test data set and could not be evaluated.

Future studies can prospectively evaluate the actual deployment of this software in an EMS system in terms of both quantitative and qualitative aspects for both audits and paramedics. Machine learning models used for named entity recognition need to be recalibrated over time to reflect changes in the documentation practices of practitioners over time and changes in personnel over time. The evaluation of such changes over time could be the focus of future studies. Finally, the evaluation of the named entity recognition model on data from other EMS systems will help to determine if the performance we observed is generalizable.

## 5. Conclusions

In this proof-of-concept study, we demonstrated the process of developing a reliable NER model that could reliably identify clinical entities from unlabeled paramedic free text reports. This model can be used in an EMS clinical audit system that automates the audit process. It allows us to increase the proportion of cases that undergo auditing to complete coverage, even as we experience increasing demand for EMS services in Singapore, while reducing mental fatigue for human auditors.

## Figures and Tables

**Table 1:** Three example text reports under three different case categories before and after pre-processing. The ground truth entities are bolded and underlined.

Category	Original Report	Pre-Processed Report
Acute coronary syndrome	hx from pt c/o chest pain x 2/7 crushing in nature, non-radiating. no trauma. no fall. o/a pt was sitting, alert, conscious. pt was gtn 1 tab by sn. o/e pt not pallor or diaphoretic. no sob/ giddiness/ nausea/ vomitting. afebrile. given 300 mg aspirin stat dose & 1 gtn spray 0.4 mg with total relieved. 12 lead ecg done: sinus rhythm. no other medical complaints	hx from pt c o chest pain x 2/7 crushing in nature non radiating no trauma no fall o a pt was sitting alert conscious pt was **gtn** 1 tab by sn o e pt not pallor or diaphoretic no sob giddiness nausea vomitting afebrile given 300 mg **aspirin** stat dose & 1 **gtn spray** 0 4 mg with total relieved **12 lead ecg** done **sinus rhythm** no other medical complaints
Stroke	hx from helper, @9 m noted pt turns lethargic, but able to enunciate words clearly, @ 12 pm, noted pt slurred speech w slight rt facial droop. @2 pm, tried to feed pt water, and noted dysphagia, drooling. went to see gp @ 310 pm, noted to send to a&e. o/a, pt sitting, gcs 15, slight dementia. no c/o unwell. o/e, noted slight rt facial droop+ slurred speech. no bilateral weakness. pt is off hypertension med for a long time. usual bp @ 115/57	hx from helper 9 m noted pt turns lethargic but able to enunciate words clearly 12 pm noted pt **slurred speech** w slight rt **facial droop** 2 pm tried to feed pt water and noted dysphagia drooling went to see gp 310 pm noted to send to a&e o a pt sitting gcs 15 slight dementia no c o unwell o e noted slight rt **facial droop** **slurred speech** no bilateral weakness pt is off hypertension med for a long time usual bp 115 57
Bleeding	o/a- pt sitting conscious alert. hx fr pt- pt fell due to slippery floor, unsure hit what object noted bleeding, no loc. o/e- noted 3 cm laceration active bleeding. noted dislocated rt shoulder, pt claimed numbness but is due to fall 2/12 ago, did not see dr. pt unable to give furthur hx as he does not wish to talk much.	o a pt sitting conscious alert hx fr pt pt fell due to slippery floor unsure hit what object noted **bleeding** no loc o e noted 3 cm laceration **active bleeding** noted dislocated rt shoulder pt claimed numbness but is due to fall 2 12 ago did not see dr pt unable to give furthur hx as he does not wish to talk much

**Table 2:** Overview of the named clinical entities in our dataset. Around 2.7% of the tokens are associated with an entity, and the rest are “O” tokens.

Category	Entity	Training				Development				Test			
		Entiti-es	% of Total	Tokens	ATE *	Entiti-es	% of Total	Tokens	ATE *	Entiti-es	% of Total	Tokens	ATE *
Clinical Procedure	ECG	26,688	50.6%	43,661	1.64	691	49.1%	1160	1.68	665	48.9%	1101	1.66
Clinical Procedure	Stroke Assessment	6571	12.5%	10,102	1.54	175	12.4%	273	1.56	200	14.7%	306	1.53
Clinical Procedure	Intravenous Cannulation	2054	3.9%	2559	1.25	64	4.5%	83	1.30	50	3.7%	69	1.38
Clinical Procedure	Burns Cooling	57	0.1%	57	1.00	4	0.3%	4	1.00	0	0.0%	0	NA
Clinical Procedure	Valsalva Maneuver	30	0.1%	44	1.47	2	0.1%	2	1.00	0	0.0%	0	NA
Clinical Finding	Bleeding	7422	14.1%	8785	1.18	189	13.4%	230	1.22	182	13.4%	218	1.20
Clinical Finding	Signs Of Obvious Death	323	0.6%	639	1.98	10	0.7%	18	1.80	8	0.6%	16	2.00
Medication	Nitroglycerin (GTN)	2648	5.0%	3835	1.45	72	5.1%	104	1.44	64	4.7%	102	1.59
Medication	Aspirin	1644	3.1%	1644	1.00	51	3.6%	51	1.00	45	3.3%	45	1.00
Medication	Normal Saline	1371	2.6%	4206	3.07	41	2.9%	115	2.80	44	3.2%	135	3.07
Medication	Penthrox	568	1.1%	568	1.00	19	1.3%	19	1.00	14	1.0%	14	1.00
Medication	Dextrose/ Glucose	447	0.8%	447	1.00	13	0.9%	13	1.00	14	1.0%	14	1.00
Medication	Adrenaline	412	0.8%	412	1.00	14	1.0%	14	1.00	8	0.6%	8	1.00
Medication	Diazepam	394	0.7%	394	1.00	8	0.6%	8	1.00	11	0.8%	11	1.00
Medication	Salbutamol	1794	3.4%	1977	1.10	50	3.6%	57	1.14	48	3.5%	55	1.15
Medication	Tramadol	310	0.6%	310	1.00	5	0.4%	5	1.00	7	0.5%	7	1.00
Medication	Syntometrine	45	0.1%	45	1.00	0	0.0%	0	NA	1	0.1%	1	1.00
Total (% of Total)		52778 (100%)		79685 (2.73%)		1408 (100%)		2156 (2.81%)		1361 (100%)		2102 (2.74%)	

* ATE: Average number of Tokens per Entity.

**Table 3:** Entity-level performance of our NER model on the test set. All three models show indistinguishably excellent performance of F1 scores.

Evaluation Mode	Model	MUC-5 Scoring						SemEval’13 Metrics		
		COR	INC	MIS	SPU	POS	ACT	Precision	Recall	F1-Score
Entity Type Matching	BiLSTM-CRF	1336	0	25	26	1361	1362	0.981	0.982	0.981
	BERT_BASE_	1343	0	20	31	1363	1374	0.977	0.985	0.981
	ClinicalBERT	1343	0	20	30	1363	1373	0.978	0.985	0.982
Strict Evaluation	BiLSTM-CRF	1329	7	25	26	1361	1362	0.976	0.976	0.976
	BERT_BASE_	1335	8	20	31	1363	1374	0.972	0.979	0.976
	Clinical-BERT	1334	9	20	30	1363	1373	0.972	0.979	0.975

**Table 4:** Comparison of model complexity and inference speed. Inference wall time is reported using the mean and standard deviation of wall clock time over 100 iterations of predicting a sample sentence, without the overhead of loading libraries and the model itself.

	Model Parameters (in Millions)	Model Checkpoint Size (Mb)	Inference Wall Time (ms)	
BiLSTM-CRF	3.83	15	40	(7.5)
BERT_BASE_	109.50	418	274	(16.2)
Clinical-BERT	108.33	414	286	(25.6)

## Data Availability

The data supporting the findings of this study are available from the corresponding author upon reasonable request, subject to approval by SCDF.

## Supplementary Materials

The following are available online at https://www.mdpi.com/article/10.3390/ijerph18157776/s1, supplementary file.
