# Supplementary material for: A Weakly-Supervised Named Entity Recognition Machine Learning Approach for Emergency Medical Services Clinical Audit
Source: Int J Environ Res Public Health. 2021 Jul 22;18(15):7776. doi: 10.3390/ijerph18157776 (PMC8345494; doi:10.3390/ijerph18157776)
Supplement: Supplementary file 1 [file ijerph-18-07776-s001.zip › ijerph-1303187-supplementary.pdf]

Supplementary A

The audit workflow in our system is depicted in Appendix Figure 1. First, we determine the clinical scenario based on some eligibility criteria. These eligibility criteria may be present in structured fields such as "Chief complaint" or they might be present in unstructured free text such as "Physical finding". Information contained in structured fields can be accessed by simple Boolean statements. Information contained in free text is extracted by a named entity recognition model. Gathering information from both sources, we obtain the scenario type and the paramedic actions performed for a case. For each scenario type, the correct actions are actually protocoled, as shown in Appendix Table 1. For example, the use of sublingual nitroglycerin for acute coronary syndrome is contraindicated in patients who have systolic blood pressure less than 90mmHg. The paramedics actions are compared to the protocol actions to check if the protocol actions were performed or attempted when indicated. For the purposes of the clinical performance audit, documentation of an attempted action, such as a failed attempt to insert an intravenous cannula or offering a medication is sufficient to generate a pass.

The results of the comparison are then used to compute summary statistics in the form of an audit report which can contain data aggregated at multiple levels. At a case-level, a report can be generated to indicate which actions were performed for a particular incident. At a provider-level, the results can also be aggregated to calculate the frequency for which indicated actions were performed for all cases attended to by a particular provider. At a system-level, the frequency of indicated actions for various clinical scenarios can be calculated to indicate overall performance.

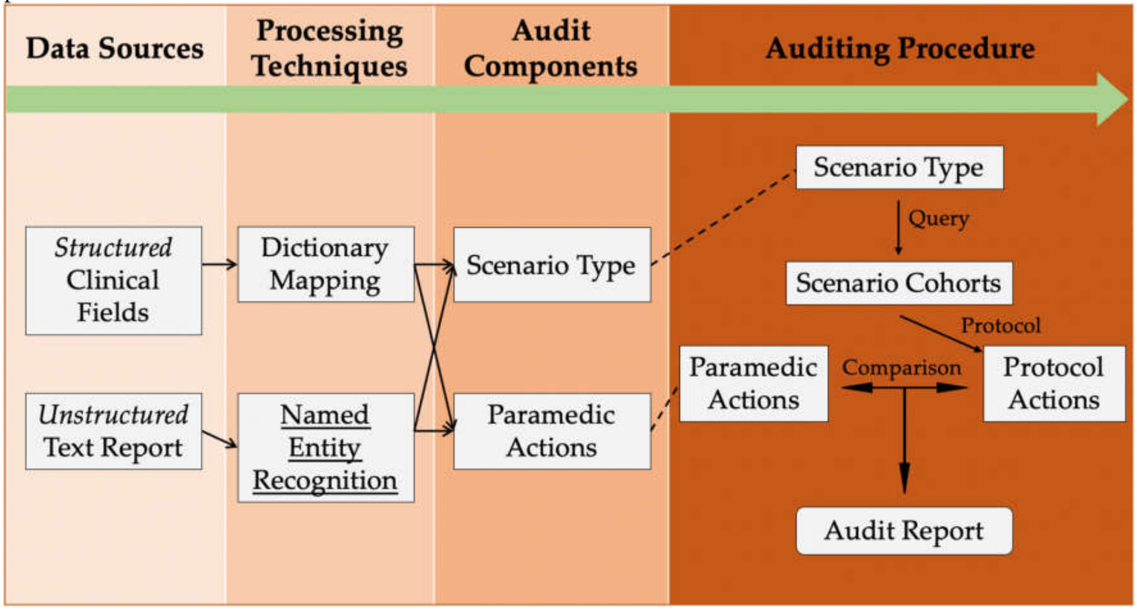

Supplementary Figure S1. System architecture and process flow of our EMS audit system.

Supplementary Table S1. Three types of clinical categories, the underlying clinical scenarios for audit and the protocol actions for each scenario. For "acute coronary syndrome" and "bleeding patient", additional actions are required if the systolic blood pressure of the patient is above or below a certain threshold.

Clinical Category	Scenario Type	Protocol Action
**Acute Coronary Syndrome**<br>Eligibility criteria:<br>- Chief complaint of "Chest Pain"	Default	•Oral aspirin 300mg given<br>•12 lead electrocardiogram performed
	+ Systolic blood pressure ≥90mmHg	Additional action:<br>•Sublingual nitroglycerin given
**Stroke**<br>Eligibility criteria:<br>- Chief complaint of "Suspected Stroke"	Default	•Cincinnati prehospital stroke scale performed and recorded<br>•Capillary blood glucose recorded
**Bleeding Patient**<br>Eligibility criteria:<br>- Physical finding of "Active Bleeding"	Default	•Bleeding control applied (e.g. dressing, manual compression, tourniquet)
	+ Systolic blood pressure <80mmHg	Additional actions:<br>•IV access established<br>•IV normal saline administered

Supplementary B

Appendix Table S2 shows the performance of the BiLSTM-CRF model over the token classes on the development set and test set. It achieves a weighted average F1 score of 0.963 on the development set and 0.980 on the test set.

Supplementary Table S2. Token-level performance of our NER model on the development and test set.

Token	Developmernt Set				Test Set			
	Precision	Recall	F1-Score	Support	Precision	Recall	F1-Score	Support
B-FINDING_BLEEDING	0.930	0.979	0.954	189	0.978	0.989	0.984	182
B-FINDING_OBVIOUSDEATH	1.000	0.800	0.889	10	1.000	0.875	0.933	8
B-MEDICATION_ADRENALINE	1.000	0.857	0.923	14	1.000	1.000	1.000	8
B-MEDICATION_ASPIRIN	1.000	1.000	1.000	51	1.000	0.978	0.989	45
B-MEDICATION_DEXTROSE	1.000	1.000	1.000	13	1.000	1.000	1.000	14
B-MEDICATION_DIAZEPAM	1.000	1.000	1.000	8	1.000	1.000	1.000	11
B-MEDICATION_GTN	1.000	1.000	1.000	72	0.955	1.000	0.977	64
B-MEDICATION_NORMALSALINE	0.895	0.829	0.861	41	0.930	0.909	0.920	44
B-MEDICATION_PENTHROX	1.000	0.947	0.973	19	1.000	1.000	1.000	14
B-MEDICATION_SALBUTAMOL	1.000	1.000	1.000	50	0.978	0.938	0.957	48
B-MEDICATION_SYNTOMETRINE	0.000	0.000	0.000	0	1.000	1.000	1.000	1
B-MEDICATION_TRAMADOL	1.000	1.000	1.000	5	1.000	1.000	1.000	7
B-PROCEDURE_BURNSCOOLING	1.000	1.000	1.000	4	0.000	0.000	0.000	0
B-PROCEDURE_ECG	0.956	0.981	0.969	691	0.986	0.986	0.986	665
B-PROCEDURE_IVCANNULA	0.953	0.953	0.953	64	0.980	1.000	0.990	50
B-PROCEDURE_STROKEASSESSMENT	0.934	0.977	0.955	175	0.975	0.975	0.975	200
B-PROCEDURE_VALSALVA	1.000	0.500	0.667	2	0.000	0.000	0.000	0
I-FINDING_BLEEDING	0.975	0.951	0.963	41	1.000	0.972	0.986	36
I-FINDING_OBVIOUSDEATH	1.000	0.875	0.933	8	1.000	0.875	0.933	8
I-MEDICATION_GTN	1.000	1.000	1.000	32	0.946	0.921	0.933	38
I-MEDICATION_NORMALSALINE	0.931	0.905	0.918	74	0.978	0.956	0.967	91
I-MEDICATION_SALBUTAMOL	1.000	0.857	0.923	7	0.857	0.857	0.857	7
I-PROCEDURE_ECG	0.998	0.947	0.972	469	0.998	0.972	0.985	436
I-PROCEDURE_IVCANNULA	0.900	0.947	0.923	19	1.000	0.947	0.973	19
I-PROCEDURE_STROKEASSESSMENT	0.931	0.959	0.945	98	1.000	0.962	0.981	106
I-PROCEDURE_VALSALVA	0.000	0.000	0.000	0	0.000	0.000	0.000	0
Weighted Average	0.964	0.964	0.963		0.985	0.975	0.980	
